# 582. Burns from Lithium-ion Batteries in the US - A Comprehensive National Analysis

**DOI:** 10.1093/jbcr/irag033.355

**Published:** 2026-04-06

**Authors:** Clifford C Sheckter, Francis T Pleban, Jennifer K Shah, Kelly Ransdell, Rebecca Coffey

**Affiliations:** Stanford University School of Medicine, Palo Alto, CA; Tennessee State University, Nashville, TN; Geisel School of Medicine at Dartmouth, NY, New York; National Fire Protection Association, Quincy, MA; Parkland Health, Dallas, TX

## Abstract

**Introduction:**

Lithium-ion cells power most personal electronics and mobility devices, delivering high energy density with risk of thermal runaway in damaged cells or misuse. Consequent flames/explosions make these batteries an emerging hazard source from thermal injuries. Despite growing reports, the epidemiology remains poorly defined since injury surveillance rarely codes lithium-ion involvement explicitly and most reports are product- or jurisdiction-specific. To address this gap, we used two complementary national datasets to quantify the national burden and characterize hospitalized cases.

**Methods:**

We performed a two-database analysis using the U.S. Consumer Product Safety Commission’s National Electronic Injury Surveillance System (NEISS), 2020-2024, and the American Burn Association’s Burn Care Quality Platform (ABA-BCQP), 2013-2022. From NEISS, burn cases were identified through the predefined injury mechanism field with the inclusion of a narrative field search. The mechanism was queried including all lithium battery operated devices with exclusions for non-lithium contexts. National weighting was specified with Stratum and Primary Sampling Unit variables. Subgroup for type of electronic was defined. The free text field for ABA-BCQP was queried for word combinations of lithium-ion batteries and devices. Inpatient demographics, injury severity, hospital course, and outcomes were described.

**Results:**

Lithium-ion battery burns increased by 64.2% from 2222 in 2020 (6.7/1000,000) to 3648 in 2024 (10.7/1000,000). Fire service involvement was documented in 33.5% of events. The most frequent device categories included phone batteries (32.9%) and electronic cigarette/vape devices (12.8%). Mean age was 38.5 years and 86.4% were male. Median total body surface area (TBSA) injured was 4.0%, with a maximum injury size of 46% TBSA. 40.4% received surgical treatment; of those undergoing surgery, there was a median of 3 operations per patient. Overall mortality was 0.8%. Payer mix included 42.0% private insurance, 20.5% Medicaid, 12.3% uninsured, and 10.0% Medicare. Racial distribution was 75.2% White, 10.0% Black, 2.2% Asian; 10.1% were Hispanic.

**Conclusions:**

Lithium-ion battery burns are increasing in the U.S., with mobile phones and electronic cigarettes/vaping as prominent drivers. While most inpatient injuries are relatively small, 40% require surgery and nearly 1% die—emphasizing the severity and the need for prevention.

**Applicability of Research to Practice:**

Americans should only use certified devices per nationally recognized testing laboratories. Burn prevention programs should include targeted messaging on lithium-ion battery risks and early signs of thermal runaway (e.g., swelling, overheating, smoke/odor), safe charging/storage, and device/charger quality—paired with surveillance efforts that explicitly code battery involvement.

**Funding for the study:**

N/A.

